# Prognostic Value of APACHE II and RTS in Predicting ICU Mortality Among Multitrauma Patients: A Single-Center Study from Turkey

**DOI:** 10.3390/jcm15176650

**Published:** 2026-08-28

**Authors:** Ahmet Düzgün, Maşallah Çakırer, Seher Yanatma

**Affiliations:** Department of Intensive Care, Gazi Yaşargil Training and Research Hospital, University of Health Sciences, Diyarbakır 21070, Türkiye; drmcakirer@gmail.com (M.Ç.); seheryanatma@hotmail.com (S.Y.)

**Keywords:** sepsis, multitrauma, intensive care unit, APACHE II, revised trauma score, mortality, prognostic model, Turkey

## Abstract

**Background/Objectives**: Trauma remains a leading global cause of mortality and morbidity, particularly in low- and middle-income countries. Multitrauma patients often require intensive care and exhibit high mortality rates. Prognostic scoring systems such as the Acute Physiology and Chronic Health Evaluation II (APACHE II) and Revised Trauma Score (RTS) are widely used to predict outcomes; however, their performance in middle-income settings like Turkey has not been well established. This study aimed to evaluate the prognostic accuracy of APACHE II and RTS in predicting intensive care unit (ICU) mortality among multitrauma patients. **Methods**: This retrospective observational study was conducted in the General ICU of Diyarbakır Gazi Yaşargil Training and Research Hospital between January 2022 and December 2024. Adult patients (≥18 years) with multitrauma involving at least two anatomical regions were included. Demographic data, clinical scores (APACHE II, RTS, Sequential Organ Failure Assessment [SOFA], Injury Severity Score [ISS], Trauma and Injury Severity Score [TRISS]), laboratory parameters, complications, and outcomes were recorded. Univariate and multivariate logistic regression analyses identified independent predictors of ICU mortality. Receiver operating characteristic (ROC) analysis assessed the discriminatory ability of prognostic scores. **Results**: A total of 145 patients were analyzed, with an ICU mortality rate of 13.1%. Non-survivors had significantly higher APACHE II (27 ± 4 vs. 13 ± 6, *p* < 0.001) and SOFA scores (7 [4–13] vs. 2 [1–6], *p* < 0.001) and lower RTS (4.2 ± 1.7 vs. 7.2 ± 1.1, *p* < 0.001). Acute kidney injury, sepsis, septic shock, and mechanical ventilation were more frequent among non-survivors (*p* < 0.01 for all). In multivariate analysis, APACHE II (odds ratio [OR]= 1.54, 95% confidence interval [CI] 1.16–2.04, *p* = 0.003) and RTS (OR = 0.36, 95% CI 0.16–0.81, *p* = 0.013) were independent predictors of ICU mortality. ROC analysis showed excellent prognostic performance for APACHE II (area under the curve [AUC] = 0.959) and RTS (AUC = 0.888). **Conclusions**: In this retrospective single-center cohort, APACHE II and RTS were independently associated with ICU mortality and demonstrated good discriminatory performance for early risk stratification in critically ill multitrauma patients. The lower mortality observed in our cohort should be interpreted cautiously, as it may also reflect differences in case-mix, injury severity, ICU admission practices, and referral patterns. These findings require external validation in larger multicenter cohorts before broader generalization. Future studies should also validate the identified cut-off values and further investigate the prognostic role of biomarkers such as procalcitonin.

## 1. Introduction

Trauma represents a major global contributor to mortality and morbidity, serving as one of the leading causes of emergency department visits, especially among young adults [1]. Optimal care for critically injured patients starts at the scene of the injury, underscoring the critical importance of pre-hospital care systems in improving survival rates [2].

According to Global Burden of Disease studies, injuries contribute to over 5 million deaths each year, comprising roughly 9% of worldwide mortality. Although earlier estimates projected that trauma-related fatalities could climb to 8.4 million by 2020, current data reaffirm trauma as a major yet preventable driver of mortality, with the greatest impact observed in low- and middle-income countries where healthcare systems often face significant resource constraints [1,3]. European trauma registries indicate that pre-hospital mortality rates substantially surpass in-hospital mortality among patients younger than 65 years, highlighting the critical need for effective early interventions and sophisticated trauma care systems [4].

Trauma frequently results in prolonged disability, diminished productivity, and a decreased quality of life for those who survive [5,6]. The prevalence and consequences of trauma differ significantly across regions, shaped by socioeconomic conditions, mechanisms of injury, and the availability of specialized trauma and critical care services [1,7].

Trauma involves a wide range of injury mechanisms, such as falls, motor vehicle collisions, penetrating and blunt injuries, electrical shocks, explosions, burns, and industrial incidents. Multitrauma, characterized by injuries to at least two major anatomical regions (head/face/neck, thorax, abdomen, or extremities), is linked to elevated mortality rates, particularly in the initial hours following injury, frequently occurring in the pre-hospital phase [7,8]. Individuals with multitrauma admitted to the intensive care unit (ICU) often exhibit severe injuries necessitating advanced organ support, with ICU mortality rates reported at 25–30%, although global trends suggest a progressive decrease [9,10].

Despite the availability of prognostic scoring systems such as APACHE II, SOFA, ISS, RTS, and TRISS, their predictive accuracy in multitrauma populations, particularly in middle-income settings like Turkey, where trauma care systems face resource constraints and regional data are scarce, remains underexplored due to limited regional data and variations in trauma care systems [11,12,13]. This gap hinders the development of tailored management strategies to optimize outcomes. This study aims to evaluate clinical and demographic risk factors associated with mortality in multitrauma patients admitted to the ICU and to assess the prognostic performance of widely used trauma scoring systems in a Turkish tertiary care setting, addressing the scarcity of region-specific evidence.

## 2. Materials and Methods

### 2.1. Study Design

This retrospective cohort study was carried out in the General Intensive Care Unit (ICU) at Diyarbakır Gazi Yaşargil Training and Research Hospital, a tertiary healthcare facility in Turkey, spanning from 1 January 2022, to 31 December 2024. The study protocol received approval from the hospital’s ethics committee (Approval Code: 586, Date: 5 September 2025) and adhered to the principles of the Declaration of Helsinki and Turkey’s Personal Data Protection Law (KVKK).

### 2.2. Participants

The study included adult patients (≥18 years) admitted to the ICU with multitrauma, defined as injuries involving at least two anatomical regions (head/neck, thorax, abdomen, pelvis, or extremities). Patients were excluded if they were younger than 18 years, had single-region trauma, sustained burn injuries, or had incomplete medical records. All consecutive eligible patients admitted during the study period who met the inclusion criteria were included in the analysis.

For the purposes of this retrospective study, multitrauma was defined as injuries involving at least two anatomical regions, consistent with the institutional trauma classification used during the study period.

### 2.3. Data Collection

Data were extracted from electronic medical records and patient files using a standardized form, anonymized, and stored securely. Variables included demographics (age, sex, comorbidities), trauma characteristics (mechanism, type, injured regions), clinical scores (Glasgow Coma Scale [GCS], Acute Physiology and Chronic Health Evaluation II [APACHE II] [14], Sequential Organ Failure Assessment [SOFA] [15], Injury Severity Score [ISS] [16], Revised Trauma Score [RTS] [17], Trauma Injury Severity Score [TRISS] [18]), laboratory parameters (complete blood count, biochemistry, arterial blood gas, C-reactive protein, procalcitonin), complications (sepsis [19], acute kidney injury [AKI] [20]), interventions (surgery, hemodialysis, blood transfusions, invasive mechanical ventilation), and outcomes (ICU length of stay, mortality). Clinical scores were calculated by two ICU specialists, with discrepancies resolved by consensus, and were selected for their established validity and widespread use in trauma and ICU settings [14,15,16,17,18]. In addition to APACHE II and RTS, SOFA, ISS, and TRISS were evaluated as complementary prognostic scoring systems to allow a broader comparison of physiological and anatomical severity assessment in critically ill multitrauma patients.

### 2.4. Statistical Analysis

Patients were stratified into survivor and non-survivor groups. Normality was assessed using the Kolmogorov–Smirnov test. Normally distributed variables are reported as mean ± standard deviation, non-normally distributed variables as median (interquartile range), and categorical variables as counts (percentages). Comparisons were performed using the independent samples *t*-test, Mann–Whitney U test, chi-square test, or Fisher’s exact test, as appropriate. The prognostic scores (APACHE II, RTS, SOFA, ISS, and TRISS) were initially evaluated individually for their associations with ICU mortality. Consistent with the primary objective of the study, APACHE II and RTS were entered into the multivariable logistic regression model. ISS and TRISS were not included in the final model because they were not significantly associated with mortality in the univariate analyses. SOFA was evaluated as a complementary measure of organ dysfunction but was not included in the final model because it was not part of the primary comparison between APACHE II and RTS. Given the limited number of mortality events, the final model was intentionally restricted to two predictors to maintain a parsimonious model and minimize the potential for model overfitting. Results are reported as odds ratios (ORs) with 95% confidence intervals (CIs). The prognostic performance of the clinical scores was assessed using receiver operating characteristic (ROC) curve analysis, including the area under the curve (AUC), sensitivity, and specificity. A two-sided *p*-value < 0.05 was considered statistically significant. All statistical analyses were performed using IBM SPSS Statistics version 29.0 (IBM Corp., Armonk, NY, USA).

## 3. Results

### 3.1. Patient Characteristics

Between 1 January 2022 and 31 December 2024, a total of 145 patients with multitrauma were admitted to the General Intensive Care Unit (ICU) at Diyarbakır Gazi Yaşargil Training and Research Hospital. The overall ICU mortality rate was 13.1% (19/145). The mean age was 55 ± 16 years, with 98 patients (68%) being male. On admission, the mean Glasgow Coma Scale (GCS) score was 12 ± 3, the Acute Physiology and Chronic Health Evaluation II (APACHE II) score was 14 ± 8, and the median Sequential Organ Failure Assessment (SOFA) score was 3 [1–6]. The mean Revised Trauma Score (RTS) was 6.7 ± 1.5, the Injury Severity Score (ISS) was 21.6 ± 16.5, and the Trauma and Injury Severity Score (TRISS) was 86.6 ± 16.1 for blunt injuries and 85.5 ± 18.9 for penetrating injuries. Comorbidities included hypertension in 41 patients (28%) and diabetes mellitus in 28 patients (19%) (Table 1).

### 3.2. Trauma Characteristics and Interventions

The predominant mechanism of injury was falls (43%), followed by motor vehicle accidents, with 32% involving passengers and 21% pedestrians (Table 2). The most frequently injured regions were the chest (55%), head and neck (53%), extremities (56%), abdomen (25%), and pelvis (22%) (Table 3). Acute kidney injury (AKI) occurred in 25% of patients, with 8% requiring hemodialysis. Surgical interventions were performed in 63% of patients, and 43% required invasive mechanical ventilation (IMV) with a median duration of 5 days [1–12]. The median ICU length of stay (LOS) was 7 days [3–13]. Sepsis was observed in 19% of patients, and septic shock occurred in 10% (Table 4).

### 3.3. Comparative Analysis

Survivors (*n* = 126) and non-survivors (*n* = 19) exhibited significant differences in several clinical parameters. Non-survivors had lower GCS scores (6 ± 3 vs. 12 ± 3, *p* < 0.001), higher APACHE II scores (27 ± 4 vs. 13 ± 6, *p* < 0.001), elevated SOFA scores (7 [4–13] vs. 2 [1–6], *p* < 0.001), and lower RTS scores (4.2 ± 1.7 vs. 7.2 ± 1.1, *p* < 0.001). Laboratory findings revealed higher procalcitonin (3.3 [0.7–7.7] vs. 0.5 [0.2–2.1] ng/mL, *p* = 0.007) and creatinine levels (1.5 ± 0.7 vs. 0.84 ± 0.33 mg/dL, *p* = 0.039) in non-survivors (Table 5). Complications were more prevalent in non-survivors, including AKI (63% vs. 19%, *p* = 0.002), sepsis (79% vs. 10%, *p* < 0.001), septic shock (68% vs. 2%, *p* < 0.001), hemodialysis requirement (31% vs. 4%, *p* = 0.002), and IMV use (95% vs. 36%, *p* < 0.001). The duration of IMV was significantly longer in non-survivors (11 [5–36] vs. 3 [1–10] days, *p* = 0.024). Non-survivors also received more fresh frozen plasma transfusions (47% vs. 15%, *p* = 0.014). No significant differences were observed in ICU LOS, comorbidities, or other laboratory parameters such as hemoglobin and C-reactive protein (*p* > 0.05) (Table 4 and Table 5).

### 3.4. Mortality Predictors

Univariate analyses identified RTS as significantly associated with mortality (*p* = 0.01), whereas ISS (*p* = 0.08) and TRISS (blunt: *p* = 0.14; penetrating: *p* = 0.13) showed no significant association. In multivariable logistic regression, APACHE II (OR: 1.54, 95% CI: 1.16–2.04, *p* = 0.003) and RTS (OR: 0.36, 95% CI: 0.16–0.81, *p* = 0.013) were independent predictors of ICU mortality. Receiver operating characteristic (ROC) curve analysis demonstrated high discriminatory power for APACHE II (AUC: 0.959, 95% CI: 0.921–0.996, *p* < 0.001) and RTS (AUC: 0.888, 95% CI: 0.805–0.971, *p* < 0.001), with optimal cut-off points of ≥18.5 (92.3% sensitivity, 84.4% specificity) for APACHE II and ≤4.8 (92.2% sensitivity, 61.5% specificity) for RTS. In contrast, SOFA, ISS, and TRISS did not exhibit significant prognostic performance (Table 1 and Table 6).

## 4. Discussion

This study evaluated mortality and risk factors in 145 multitrauma patients admitted to a tertiary ICU in Turkey between January 2022 and December 2024. The observed mortality rate of 13.1% was lower than the 25–30% reported globally [9,10]. Although this finding may reflect improvements in trauma care, it should be interpreted cautiously, as differences in case-mix, injury severity distribution, ICU admission criteria, referral patterns, and survivor selection may also have contributed to the lower mortality observed in our cohort. The relatively moderate mean Injury Severity Score (ISS) of 21.6 ± 16.5 may have further influenced these findings [1]. In addition, van Breugel et al. reported a 1.8% annual decline in trauma mortality since 1966, which they attributed to advances in pre-hospital care and intensive care management [10]. APACHE II and RTS emerged as independent predictors, with AUCs of 0.959 and 0.888, respectively, aligning with prior studies [11,12]. Optimal cut-off values (APACHE II ≥ 18.5 and RTS ≤ 4.8) demonstrated high sensitivity (92.3% and 92.2%, respectively) and specificity (84.4% and 61.5%, respectively), supporting their potential utility for early risk stratification.

TRISS and ISS were not significantly associated with mortality, consistent with findings questioning their accuracy in specific subgroups [13]. The limited predictive value of ISS may reflect its anatomical focus, which may not capture physiological derangements in ICU patients. Non-survivors had higher rates of AKI (63%), sepsis (79%), and septic shock (68%) [Table 4], corroborating previous reports [9,11,21]. Procalcitonin elevation (*p* = 0.007) in non-survivors, versus no difference in C-reactive protein (CRP) (*p* = 0.392), suggests its potential as a mortality biomarker, supported by Li et al. [22]. Comorbidities showed no significant impact, possibly due to the dominance of acute complications.

Falls (43%) and motor vehicle accidents (53%) as primary mechanisms, with chest (55%) and head/neck (53%) injuries, align with global epidemiology [1,23]. The 63% surgical intervention rate and 7-day median ICU stay may reflect improved care systems, similar to Al-Thani et al.’s 17% mortality in Qatar [24]. Pre-hospital predictors [2] and long-term outcomes [5,6] warrant further exploration in this context.

We acknowledge that consensus-based polytrauma definitions incorporating physiological parameters and injury burden have been increasingly adopted in contemporary trauma research. However, because this was a retrospective cohort study, we applied the institutional definition that was consistently used during the study period to ensure uniform patient selection.

Strengths of this study include comprehensive clinical data collection and robust statistical analyses. Limitations include its retrospective design, the relatively small number of mortality events, manual calculation of clinical scores, and the single-center setting, which may limit the generalizability of the findings. Because only 19 mortality events occurred, the multivariable logistic regression model was intentionally restricted to two prognostic scores, and the findings should therefore be interpreted with appropriate caution. In addition, because the predictive performance of APACHE II and RTS was evaluated using the same dataset in which the analyses were performed, the reported discriminatory performance may be subject to optimism bias. Although SOFA, ISS, and TRISS were evaluated as complementary prognostic scoring systems, their predictive performance should also be interpreted cautiously. Therefore, external validation in larger multicenter cohorts is warranted before these findings can be generalized. These findings support the use of APACHE II and RTS for early risk stratification in critically ill multitrauma patients and highlight the importance of monitoring AKI and sepsis. Future studies should validate the identified cut-off values and further investigate the prognostic role of biomarkers such as procalcitonin.

## 5. Conclusions

This study demonstrated that APACHE II and Revised Trauma Score (RTS) were independently associated with ICU mortality and showed good discriminatory performance (AUCs of 0.959 and 0.888, respectively) in this cohort, supporting their potential utility for early risk stratification. The observed 13.1% mortality rate, which was lower than global estimates, should be interpreted cautiously, as differences in case-mix, injury severity, ICU admission practices, and referral patterns may also have contributed to this finding. Elevated procalcitonin levels and complications such as acute kidney injury and sepsis highlight the importance of careful clinical monitoring. These findings require external validation in larger multicenter cohorts before broader generalization. Future studies should validate the identified cut-off values and further investigate the prognostic role of biomarkers in diverse trauma populations.

## Figures and Tables

**Table 1:** Demographic characteristics, clinical scores, and comorbidities of the patients.

Variables	Survivors *n* = 126	Non-Survivors *n* = 19	*p*
Demographics & Clinical Scores			
Age, years	55 ± 15	58 ± 23	0.465
Male, *n* (%)	84 (67)	14 (74)	0.512
GCS	12 ± 3	6 ± 3	0.001
APACHE II score	13 ± 6	27 ± 4	0.001
SOFA score	2 [1–6]	7 [4–13]	0.001
RTS	7.2 ± 1.1	4.2 ± 1.7	0.001
ISS	21.6 ± 14	29.3 ± 17	0.084
Comorbidities, *n* (%)			
Hypertension	33 (26)	8 (42)	0.238
Coronary artery disease	15 (12)	6 (31)	0.095
Diabetes mellitus	23 (18)	5 (26)	0.713

Abbreviations: GCS = Glasgow Coma Scale, APACHE II = Acute Physiology and Chronic Health Evaluation II, SOFA = Sequential Organ Failure Assessment, RTS = Revised Trauma Score, ISS = Injury Severity Score.

**Table 2:** Mechanism of injury of the patients.

Variables	Survivors *n* = 126	Non-Survivors *n* = 19	*p*
Falls	56 (44)	6 (31)	0.351
Motor vehicle accidents—pedestrian	26 (21)	3 (16)	0.479
Motor vehicle accidents—passenger	39 (30)	8 (42)	0.340
Penetrating injuries	5 (4)	2 (10)	0.498

Abbreviations: *n* = number.

**Table 3:** Injury regions of the patients.

Variables	Survivors *n* = 126	Non-Survivors *n* = 19	*p*
Head & Neck	65 (52)	12 (63)	0.631
Chest	68 (54)	12 (63)	0.740
Abdomen	30 (24)	7 (37)	0.318
Pelvis	26 (21)	6 (32)	0.480
Extremity	74 (59)	7 (37)	0.165

Abbreviations: *n* = number.

**Table 4:** Renal status, treatments, and outcomes of the patients.

Variables	Survivors *n* = 126	Non-Survivors *n* = 19	*p*
Renal & Surgical status			
ARF on admission (KDIGO)	24 (19)	12 (63)	0.002
Hemodialysis requirement	5 (4)	6 (31)	0.002
Surgery	86 (68)	6 (31)	0.014
Treatment & Outcomes			
Sepsis	13 (10)	15 (79)	0.001
Septic shock	2 (2)	13 (68)	0.001
Invasive mechanical ventilation	45 (36)	18 (95)	0.001
Duration of mechanical ventilation, days	3 [1–10]	11 [5–36]	0.024
Red blood cell transfusion	44 (35)	11 (57)	0.070
Fresh frozen plasma	19 (15)	9 (47)	0.014
LOS in ICU, days	7 [2–13]	10 [6–36]	0.183

Abbreviations: ARF = acute renal failure; KDIGO = Kidney Disease: Improving Global Outcomes; LOS = length of stay; ICU = intensive care unit.

**Table 5:** Selected laboratory findings of the patients.

Variables	Survivors *n* = 126	Non-Survivors *n* = 19	*p*
CRP (mg/L) (first day)	41 [19–109]	71 [19–163]	0.392
Procalcitonin (ng/mL)	0.5 [0.2–2.1]	3.3 [0.7–7.7]	0.007
WBC (10^3^/µL)	13.6 ± 5.4	16.2 ± 8.2	0.197
Hb (g/dL)	11.5 ± 2.4	11.5 ± 2.2	0.941
Platelet (10^3^/µL)	232 ± 91	297 ± 130	0.371
Creatinine (mg/dL)	0.84 ± 0.33	1.5 ± 0.7	0.039
Lactate (mmol/L)	2 ± 1.2	3 ± 2.2	0.133
Potassium (mmol/L)	4.1 ± 1.1	4.9 ± 1.2	0.072

Abbreviations: CRP = C-Reactive Protein, WBC = White Blood Cell, Hb = Hemoglobin, g/dL = gram per deciliter, mg/dL = milligram per deciliter, mmol/L = millimole per liter.

**Table 6:** Multivariate logistic regression and ROC analysis.

Variables	OR (95% CI)	*p*	AUC (95% CI)	Cut-Off	Sensitivity (%)	Specificity (%)
APACHE II	1.54 (1.16–2.04)	0.003	0.959 (0.921–0.996)	≥18.5	92.3	84.4
RTS	0.36 (0.16–0.81)	0.013	0.888 (0.805–0.971)	≤4.8	92.2	61.5

Abbreviations: OR = Odds Ratio, CI = Confidence Interval, AUC = Area Under the Curve, APACHE II = Acute Physiology and Chronic Health Evaluation II, RTS = Revised Trauma Score. *p* < 0.05 was considered statistically significant.

## Data Availability

The datasets used and/or analyzed during the current study are available from the corresponding author upon reasonable request.

## Abbreviations

Abbreviation	Definition
AKI	Acute Kidney Injury
APACHE II	Acute Physiology and Chronic Health Evaluation II
AUC	Area Under the Curve
CI	Confidence Interval
CRP	C-Reactive Protein
GCS	Glasgow Coma Scale
ICU	Intensive Care Unit
IMV	Invasive Mechanical Ventilation
ISS	Injury Severity Score
IQR	Interquartile Range
KDIGO	Kidney Disease: Improving Global Outcomes
LOS	Length of Stay
OR	Odds Ratio
ROC	Receiver Operating Characteristic
RTS	Revised Trauma Score
SOFA	Sequential Organ Failure Assessment
TRISS	Trauma and Injury Severity Score
